# Supplementary material for: Genetic Diversity and Structure for Conservation Genetics of Goldeye Rockfish Sebastes thompsoni (Jordan and Hubbs, 1925) in South Korea
Source: Biology (Basel). 2025 Nov 6;14(11):1559. doi: 10.3390/biology14111559 (PMC12650271; doi:10.3390/biology14111559)
Supplement: Supplementary file 1 [file biology-14-01559-s001.zip › Supplementary information.pdf]

Table S1. Sampling sites and number of individuals in the study

Population ID	Location	*N*	Location	Sample date	Sample storage
BS	Busan	34	35°04'31″ N, 129°07'28" E	2018.06	Ethanol 99%
SA	Sinan	19	34°49'56″ N, 126°17'17" E	2018.06	Ethanol 99%
TY	Teongyeong	30	34°50'10″ N, 128°26'33" E	2018.06	Ethanol 99%
UL	Ulleungdo Island	35	37°31'42″ N, 130°49'37" E	2018.06	Ethanol 99%
YD	Yeongdong	35	36°32'26″ N, 129°27'05" E	2018.06	Ethanol 99%

N: Number of samples.

Table S2. Information on microsatellite markers used in genetic analysis of *S. thompsoni*

Locus (Label)	Primer sequence (5'– 3')	Ta (°C)	Allele size range (bp)	Population				Genbank acession no.	Reference
				N_A	H_O	H_e	P_{HWE}		
Sth3A	F-ATGGTGACAAGCTAGCAGTGCATTC<br>R-GACAATGTCCATCTAGGCATGACTG	56	113-121	5	0.60	0.67	0.29	AB033424	Sekino et al., 2000
Sth24	F-AGGACAGGATGTGCCCTTTTACCA<br>R-GCCTCAGAGGCCGATTTCCTTATT	56	133-139	4	0.46	0.53	0.41	AB033426	
Sth37	F-TACAGGAAACAAGACCACGGGTACAG<br>R-GCAACATCCCTTTAAGTCACCTGCAG	57	221-243	10	0.88	0.88	0.28	AB033427	
Sth45	F-CTGGGACCTAGCCTGATTACAGCA<br>R-AAACTCAGCGACAGCAGACCACA	57	192-212	10	0.71	0.76	0.50	AB033428	
Sth91	F-TTTCGATATGCTTCGCTAGGGTGTT<br>R-CCATCAAACTGCACCAACAAAGACA	57	216-220	3	0.30	0.30	0.19	AB033431	
KSs2A	F-CCCATAGCCTTGTTTACCT<br>R-TTTTGGTTATTGTCTTGGTTT	55	110-166	16	0.90	0.88	0.9837	EF109802	An et al., 2009
KSs6	F-TGCCCAGATACATTTACACAC<br>R-TAACCCCCACCCACAC	57	140-160	10	0.67	0.78	0.1779	EF109805	

Table S3. Null allele estimates, stutter diagnostics, and FreeNA ENA-corrected vs. original F_{ST} for seven microsatellite loci

Locus	Max. null-allele frequency (across 5 pops)	F_{ST} original	F_{ST} ENA corrected	ΔF_{ST} (ENA – original)
Sth3A	0.021	0.005714	0.005705	-0.000009
Sth24	0.019	0.001895	0.002258	0.000363
Sth45	0.000	0.003992	0.003992	0.000000
KSs6	0.013	0.003683	0.003555	-0.000128
KSs2A	0.000	0.008104	0.008104	0.000000
Sth91	0.000	-0.000074	-0.000075	-0.000001
Sth37	0.000	0.000973	0.000973	0.000000
All loci	0.021	0.003957	0.003970	0.000013

Max. null-allele frequency: Estimated with the EM algorithm (Dempster et al. 1977); highest frequency observed among five populations, F_{ST} ENA corrected: Weir & Cockerham's (1984) θ. ENA-corrected values computed in FreeNA (Chapuis & Estoup 2007), ΔF_{ST}: Difference between ENA-corrected and original F_{ST} (positive values indicate an increase after correction).

Table S4. Bayesian estimates of posterior mean migration rates among the five populations inferred with MIGRATE-n

	BS	SA	TY	UL	YD
BS	-	9.40	13.87	13.70	10.70
SA	10.40	-	5.00	13.10	7.04
TY	11.02	8.92	-	4.14	5.75
UL	14.94	15.31	14.33	-	15.97
YD	12.37	10.44	19.11	10.15	-
